# Artificially selecting microbial communities: If we can breed dogs, why not microbiomes?

**DOI:** 10.1371/journal.pbio.3000356

**Published:** 2019-08-30

**Authors:** Flor I. Arias-Sánchez, Björn Vessman, Sara Mitri

**Affiliations:** Department of Fundamental Microbiology, University of Lausanne, Lausanne, Switzerland

## Abstract

Natural microbial communities perform many functions that are crucial for human well-being. Yet we have very little control over them, and we do not know how to optimize their functioning. One idea is to breed microbial communities as we breed dogs: by comparing a set of microbiomes and allowing the best-performing ones to generate new communities, and so on. Although this idea seems simple, designing such a selection experiment brings with it many decisions with surprising outcomes. Xie and colleagues developed a computational model that reveals this complexity and shows how different experimental design decisions can impact the success of such an experiment.

## Abstract 2

Microbial communities provide key ecosystem services that could be improved using artificial selection. This Primer explores recent work showing that despite its potential, this approach brings with it a number of important challenges.

*“The most difficult problems in human life cannot be solved by single individuals and require coordinated teams of specialists*.*”* [1]

Selective breeding has been practiced by humans for thousands of years to produce animals and plants with desirable features. This involves comparing a set of similar organisms, selecting those with the most desirable traits, and ensuring that they produce more offspring. Yet some functions that we desire cannot be performed by individual organisms and, instead, require a group of specialists to work together. The question, then, is whether we can selectively breed whole groups or even communities composed of multiple species to improve a particular function of interest.

Selective breeding of groups of organisms—sometimes also referred to as artificial group selection or experimental group selection—has been tried before with, in some cases, impressive success. One of the first such experiments was performed on chickens [2]. Chickens are often kept in cages containing multiple birds, which has been found to improve feeding efficiency and reduce disease spread. The problem is that hens can become extremely aggressive in groups, resulting in high mortality rates. The proposed solution was to select the offspring of whole groups based on a combination of total egg production and survival rates and repeat this over multiple generations. Over time, bird numbers and survival rates went up, and aggression between them went down [3].

Groups of chickens are still composed of relatively similar organisms, however. Can we push the principle further and select for whole ecosystems composed of different species? Ecosystems that are small enough in volume to be amenable to such artificial selection experiments are microbial communities composed of bacteria, fungi, archaea, and viruses. When living in association with animals, plants, or the environment, these are often called “microbiomes.” As we learn more about the important role of microbiomes in health and disease, in food production, or in the breakdown of toxic soil pollutants, the idea of breeding them is becoming highly attractive and has been attempted a number of times over the last 2 decades [4,5]. The principle is to incubate a number of similar microbial ecosystems in parallel, allowing populations of different species to grow for some time, rank them according to a desired trait value, and then select the best communities to seed new ecosystems in a new round of selection (see Fig 1) [4].

**Fig 1:**
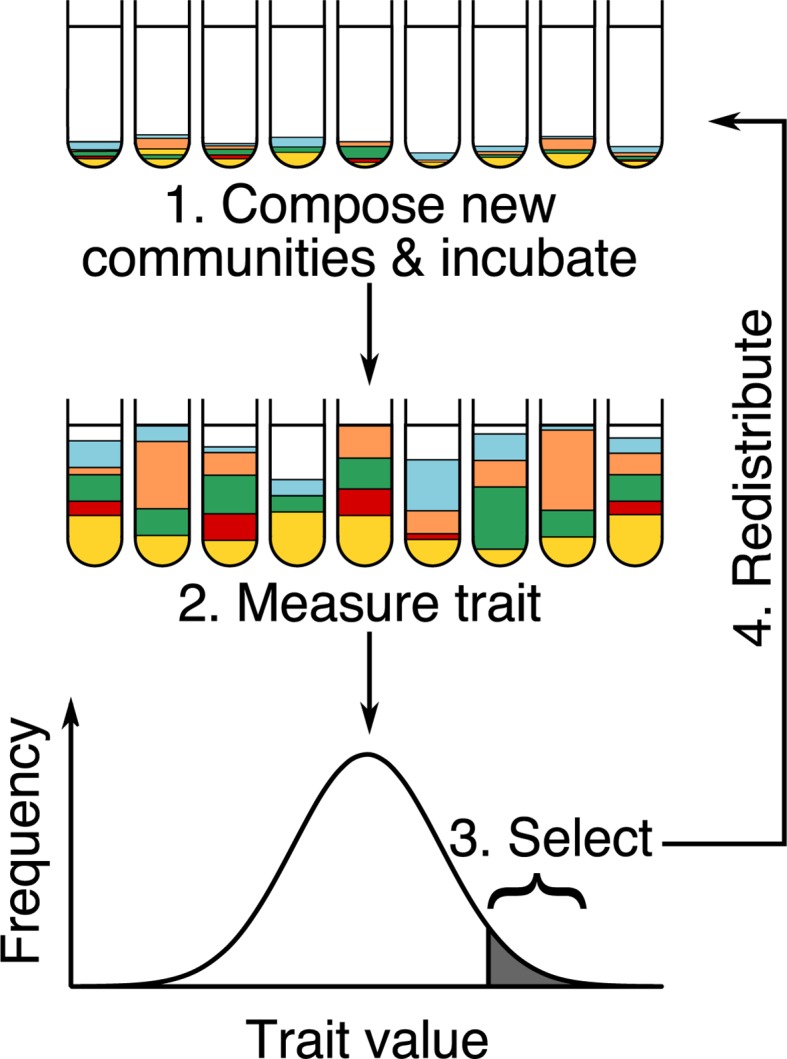
Multiple microbial communities are created and incubated in parallel (colors represent different species or lineages, and white space represents cell-free growth medium) for a given amount of time as cells grow and divide. Then, the trait value of each ecosystem is measured, communities are ranked, and the best communities are redistributed at a reduced initial population size to make a new selection round. Communities do not necessarily start with the same biomass or reach the same biomass after incubation. There are many ways to implement selection and redistribution. *Figure adapted from [6]*.

One of the first studies to use this approach involved artificially selecting soils for their ability to increase plant biomass [1], and it was quickly followed up by a study on 3-chloroaniline biodegradation using planktonic bacterial communities from ponds [6]. Both studies indicated that community selection was better than a control. Their results were not entirely conclusive, however, as the strength of these traits fluctuated significantly across selection rounds and did not work in all experimental repeats. Since then, in addition to theoretical work [7], experiments have aimed to optimize microbial communities originating from a water treatment plant for low CO_2_ production [8] and soil microbiomes for early versus late plant flowering time [9]. These studies have also been reasonably successful, but there is clearly much room for improvement.

There are a number of reasons why microbial community selection might be less effective than individual selection or group selection in organisms of a single species, despite the superficial similarity in the approach. For one thing, compared with individual selection, in which a selected unit has a "population" size of 1, microbial communities contain many different species, each potentially composed of different lineages, and huge population sizes. Individuals within these communities are likely to compete with one another. Second, cells of different lineages in a microbial ecosystem replicate at different rates, as opposed to genes within a single multicellular individual. This means that as populations grow within a selection round, the relative frequencies of lineages change over time, whereas genes within an individual mostly maintain their relative copy numbers. Third, a selection round contains many microbial generations, meaning that the length of a selection round and the mode of community propagation and redistribution are design decisions rather than well-defined features of the organism under selection. Finally, desirable community traits typically correlate with biomass, but the ability of a lineage to generate biomass will not necessarily mean that it has a high per-cell trait value. The consequences of these issues for artificial community selection are outlined in Box 1.

**Box 1. Challenges to artificially evolving microbial communities.**

Selection experiments require the propagation of the best communities to the next round based on the evaluation of the trait of interest (as in Fig 1). The success of a community selection experiment relies on inheritance of lineages making a large contribution to the trait of interest, but numerous sources of potential conflict can emerge within bacterial communities and affect the outcome of selection (Fig 2): (1) If ecological dynamics are unstable, we can expect some lineages to go extinct. This can occur through neutral drift, but it is particularly likely and troublesome if the trait of interest is costly and reduces its carrier’s growth rate relative to other community members. Conversely, if the trait leads to faster growth, single lineages may take over the population, resulting in a monoculture rather than a community [5]. (2) If the trait of interest is costly, we may also expect mutants to emerge that do not express the trait. These free riders would outcompete their ancestral lineage, and the overall trait value of the community would decrease. (3) When the length of a selection round is too short or the propagated biomass too low, growth within a selection round may not be sufficient to maintain biomass over time. (4) Similarly, if biomass correlates with the trait of interest, communities within the same selection round that have reached a high biomass will have a higher trait value even if they do not have high per-cell trait values. Selection will therefore act on growth rates and yield rather than per-cell traits as desired. (5) The trait of interest may be the result of interactions between community members. If communities are mixed in each round, important interaction partners may be decoupled from one another and reduce the community trait value [5,18]. Although these conflicts are illustrated separately, it is probable that many of them would be present at once, adding extra layers of complexity to a single experiment.

**Fig 2:**
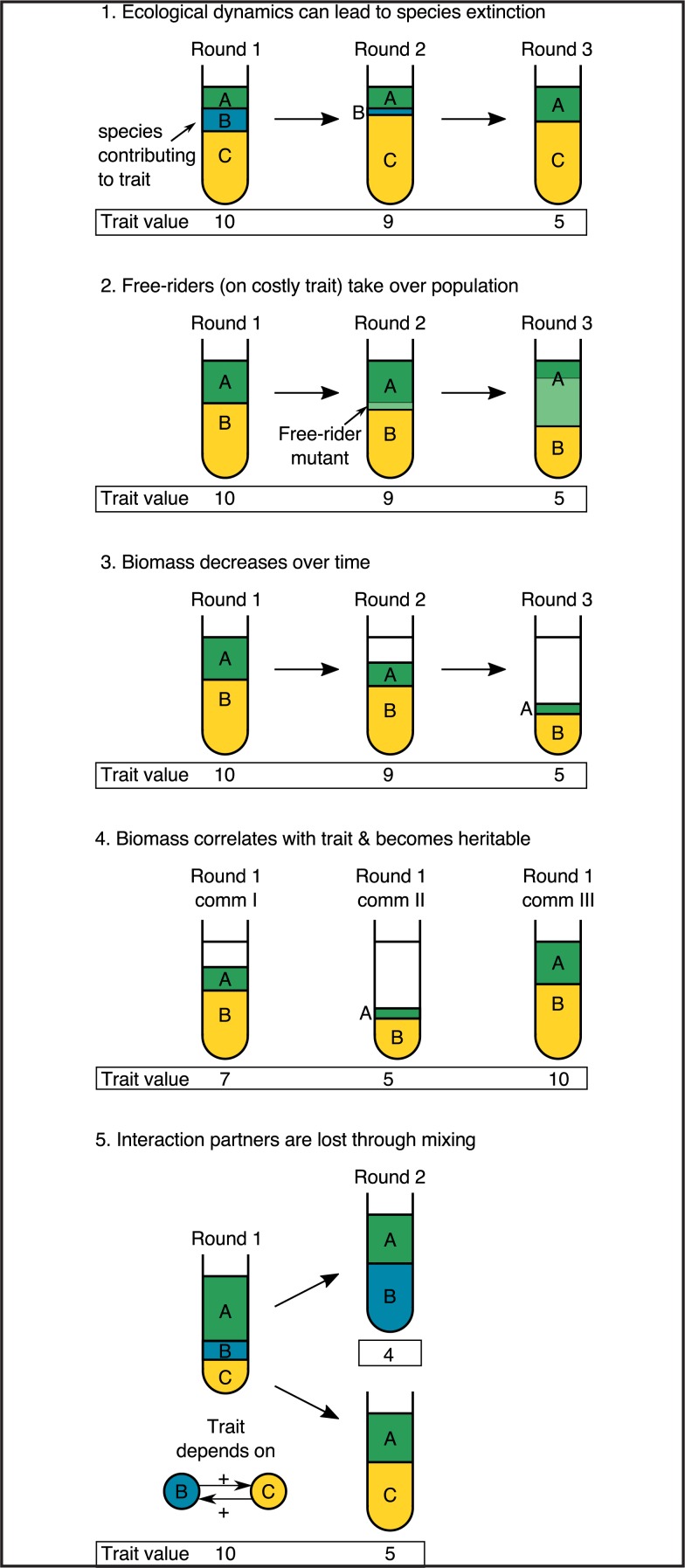
Illustration of the problems associated with microbial community selection. Each tube contains a community. Problems 1, 2, 3, and 5 show sequential rounds of selection, whereas problem 4 shows different communities within the same selection round. Letters represent species, and colors represent lineages (the two shades of green species A represent an ancestor and a mutant strain). Below each tube in a box is a trait value, which is used to select the best communities to be transmitted to the next selection round. Problems are described in Box 1.

These differences highlight that a community selection experiment involves a number of important design decisions: How many individuals should seed a starting population? How long should a selection round last? Should communities be mixed at the end of every selection round (analogous to sexual reproduction in multicellular organisms) or kept separate? How should they be replicated? In addition, the standard decisions involved in all selective breeding need to be made: What proportion of the best communities should be selected? How many communities should one evaluate and compare with one another? And so on. These experimental design decisions are bound to affect the efficacy of artificial community selection, but we do not yet understand how. Given the vast number of choices to be made, computational models offer a great way to explore what works and how to overcome the issues listed in Box 1.

This is what Xie and colleagues [10] set out to do. They model a simple community composed of two microbial species, one of which takes up a substrate to produce a by-product that the second species transforms into a final product that is desirable but costly for the microbes to produce (see Fig 3A). The goal of the selection experiment is to evolve a two-species community that is highly efficient at transforming substrate into product. By keeping the problem simple, most issues are avoided: the community is naturally quite stable—as long as the first species does not grow too quickly, the two species always converge to the same relative frequency. This avoids the problem of unpredictable ecological dynamics (problem 1 in Box 1). Second, to avoid the problem of variations in community biomass (problems 3 and 4 in Box 1), the authors standardize biomass at the beginning of each selection round (same initial population size in Fig 1 step 1). Finally, because the system is composed of only two species and population sizes of each are very large at equilibrium, it is unlikely that the species will be separated from one another on propagation, avoiding the interaction decoupling problem (problem 5 in Box 1).

**Fig 3:**
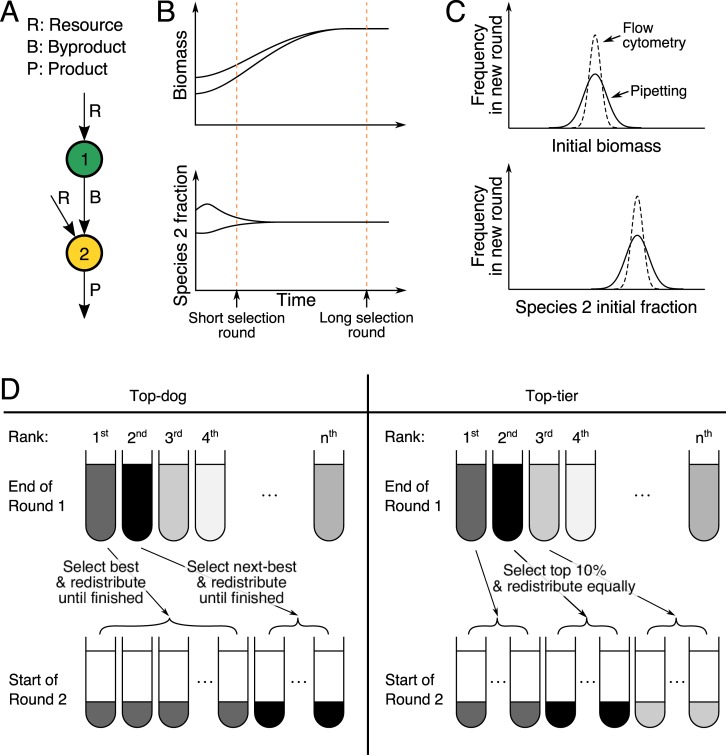
Xie and colleagues optimize community selection. (A) Their system is as follows: species 1 takes up resource R and makes by-product B, whereas species 2 takes up R and B to make the product of interest P. (B) For this system, the amount of product P a community produces is a function of biomass concentration: a community starting with greater biomass will make more product if selection rounds are short. Lengthening the selection round makes sure a community is evaluated by how much its cells produce individually rather than by how much biomass it has accumulated. Similarly, the initial fraction of species 2 affects community score if one selects early but not late. (C) Because of noise in initializing new communities, communities vary in their initial biomass and the fraction of species 2. The plots show the distribution of communities in a given round. Using flow cytometry reduces the noise compared with pipetting. Less noise means less bias in the resulting community scores because of these initial states. (D) Two selection methods were compared. In “top-dog” selection, the best-scoring community from the previous round is distributed among the fresh tubes of the next round until no cells are left, then the next best community is distributed, etc. The best community is highly favored. The “top-tier” selection method is less stringent because all top 10% of communities are equally distributed among the tubes of the next round. This second selection method worked better in Xie and colleagues’ model.

This leaves one remaining problem in their setup: the invasion of free riders that do not make the costly product (problem 2 in Box 1). They find that this problem occurs when mutations result in free riders that cannot be excluded because of noise in evaluating communities. If community scoring were perfect, the presence of even a few free riders would always lead to lower scores than in their absence, but noise can mask this negative effect. Their simulations are noisy because they mimic stochastic variations naturally arising from pipetting in a real experiment. Small variations in biomass or in species frequencies between the communities at the beginning of each selection round can have important consequences: if a community containing a small proportion of free riders (lower per-cell productivity) begins with a slightly larger biomass, at the time of selection it may have a higher overall productivity than a purely productive community and a greater likelihood of being selected for the next round. This weakens the heritability of per-cell productivity and favors cells that can quickly generate biomass. Much of the exploration of the paper then involves understanding how to make selection powerful enough to favor high per-cell productivity and to disfavor free riders or less-powerful producers.

They find that three aspects of experimental design can help to maintain selection for high per-cell productivity in communities. Essentially, all three of these involve overcoming the noise in measuring community productivity. Their first solution is to make selection rounds longer. Even if cells start with slightly different initial biomass or relative frequencies, they end up being very similar after they have consumed all the resources in the environment (equal final biomass in Fig 1 step 2). As a result, any detected differences in community productivity can be attributed to differences in per-cell productivity rather than growth rate (Fig 3B). Making a selection round too long, however, could promote the establishment of free riders, among other issues. Second, the authors simulate pipetting versus cell counting through flow cytometry and conclude that cell counting reduces variation between initial population sizes and species frequencies, leading to better heritability of per-cell productivity (Fig 3C). Third, they make selection less stringent, such that not only the very best communities are transmitted to the next round. Using their “top-tier” selection approach, highly productive but “unlucky” communities that may have been misevaluated because of a lower starting biomass, for example, are not immediately eliminated (Fig 3D).

The work by Xie and colleagues is important and very timely. As we continue to understand and appreciate the role microbiomes play in our lives, interest in improving their functions in health, bioremediation, or the synthesis of chemical compounds using selection experiments is likely to increase. Although Xie and colleagues’ work may come across as somewhat dense and theoretical, experimentalists interested in conducting microbial community selection experiments are encouraged to carefully consider their findings. Their study helps to appreciate the complexity of these experimental design issues. For example, knowing how experimental parameters such as the length of a selection round or the mode of community propagation affects the reliability of selection can avoid “mistakes” that could be very costly given how long such evolutionary experiments can be.

That said, by revealing the complexity of the problem, Xie and colleagues also make us realize that much more theoretical work needs to be done to understand artificial selection of communities. Just to name one issue, natural microbiomes contain orders of magnitude more species than the two they simulate, with unknown interactions and unpredictable ecological dynamics. We still know little about how to ensure community stability in a way that will allow us to select for a trait of interest (problem 1 in Box 1) [11]. Improving selection of such complex communities is therefore expected to be extremely challenging. Xie and colleagues have nevertheless taken a first step in the right direction and paved the way to make this possible.

Community selection experiments not only are interesting from an applied perspective but also relate to fundamental questions in ecology and evolution. For example, we see clear parallels between these artificial selection experiments and the inheritance of natural microbial communities [12]. Does natural selection act on the function of the human microbiome as it is passed from a mother to her daughter? If community-level selection is indeed such a difficult problem (Box 1), it seems unlikely that a host would manage to effectively select for advantageous microbiome functions. On the other hand, following many years of association with microbes, the mammalian gut may have evolved strategies to select for increasingly productive communities [13]. A more systematic investigation of this question is of course very timely.

A related question is whether community inheritance can favor the evolution of cooperation within communities of unrelated organisms whose interests do not naturally align. A recent artificial selection experiment was performed on a mixture of *Pseudomonas fluorescens* strains with the goal of studying the emergence of multicellularity [14], rather than optimizing community function. By selecting at the group level, the authors show that “cheats” that do not contribute to group fitness can be suppressed. Over time, their community selection method favors lineages that sacrifice their own individual fitness to the benefit of their group. Selecting for cooperative behavior between unrelated individuals is then possible in a well-controlled laboratory experiment, but whether it would evolve through natural selection is less clear. Solutions to the problems in Box 1 would be required for something like a “superorganism” with perfectly aligned interests between species in the same community to evolve naturally [15–17]. But Xie and colleagues’ study suggests that we should one day be able to artificially build such communities through carefully designed and well-controlled selection experiments. We may even dare to imagine breeding cooperative microbiomes that can be inoculated into soil to improve plant growth or used as probiotics to outcompete dysbiotic microbiomes and restore health.
